# Clinical sequencing identifies potential actionable alterations in a high rate of urachal and primary bladder adenocarcinomas

**DOI:** 10.1002/cam4.5639

**Published:** 2023-01-20

**Authors:** Melinda Varadi, Nikolett Nagy, Henning Reis, Boris Hadaschik, Christian Niedworok, Orsolya Modos, Attila Szendroi, Jason Ablat, Peter C. Black, David Keresztes, Anita Csizmarik, Csilla Olah, Nadine T. Gaisa, Andras Kiss, Jozsef Timar, Erika Toth, Erzsebet Csernak, Arpad Gerstner, Vinay Mittal, Sofia Karkampouna, Marianna Kruithof de Julio, Balazs Gyorffy, Gabor Bedics, Michael Rink, Margit Fisch, Peter Nyirady, Tibor Szarvas

**Affiliations:** ^1^ Department of Urology Semmelweis University Budapest Hungary; ^2^ Dr. Senckenberg Institute of Pathology University Hospital Frankfurt, Goethe University Frankfurt Frankfurt Germany; ^3^ Institute of Pathology, West German Cancer Center University of Duisburg‐Essen, University Hospital Essen Essen Germany; ^4^ Department of Urology, West German Cancer Center University of Duisburg‐Essen, University Hospital Essen Essen Germany; ^5^ Vancouver Prostate Centre University of British Columbia Vancouver Canada; ^6^ Department of Molecular Biology Institute of Biochemistry and Molecular Biology, Semmelweis University Budapest Hungary; ^7^ Institute of Pathology RWTH Aachen University Aachen Germany; ^8^ Department of Pathology, Forensic and Insurance Medicine Semmelweis University Budapest Hungary; ^9^ National Institute of Oncology Budapest Hungary; ^10^ Thermo Fisher Scientific Ann Arbor Michigan USA; ^11^ Department for BioMedical Research, Urology Research Laboratory University of Bern Bern Switzerland; ^12^ Department of Urology, Inselspital Bern University Hospital Bern Switzerland; ^13^ Research Centre for Natural Sciences, Cancer Biomarker Research Group Institute of Enzymology Budapest Hungary; ^14^ 2nd Department of Pediatrics and Department of Bioinformatics Semmelweis University Budapest Hungary; ^15^ Department of Pathology and Experimental Cancer Research Semmelweis University Budapest Hungary; ^16^ Department of Urology University Medical Center Hamburg‐Eppendorf Hamburg Germany

**Keywords:** molecular genetics, Oncomine, primary bladder adenocarcinoma, targeted therapy, urachal cancer

## Abstract

**Objective:**

Administration of targeted therapies provides a promising treatment strategy for urachal adenocarcinoma (UrC) or primary bladder adenocarcinoma (PBAC); however, the selection of appropriate drugs remains difficult. Here, we aimed to establish a routine compatible methodological pipeline for the identification of the most important therapeutic targets and potentially effective drugs for UrC and PBAC.

**Methods:**

Next‐generation sequencing, using a 161 cancer driver gene panel, was performed on 41 UrC and 13 PBAC samples. Clinically relevant alterations were filtered, and therapeutic interpretation was performed by in silico evaluation of drug‐gene interactions.

**Results:**

After data processing, 45/54 samples passed the quality control. Sequencing analysis revealed 191 pathogenic mutations in 68 genes. The most frequent gain‐of‐function mutations in UrC were found in *KRAS* (33%), and *MYC* (15%), while in PBAC *KRAS* (25%), *MYC* (25%), *FLT3* (17%) and *TERT* (17%) were recurrently affected. The most frequently affected pathways were the cell cycle regulation, and the DNA damage control pathway. Actionable mutations with at least one available approved drug were identified in 31/33 (94%) UrC and 8/12 (67%) PBAC patients.

**Conclusions:**

In this study, we developed a data‐processing pipeline for the detection and therapeutic interpretation of genetic alterations in two rare cancers. Our analyses revealed actionable mutations in a high rate of cases, suggesting that this approach is a potentially feasible strategy for both UrC and PBAC treatments.

## Graphical abstract

Precision oncology is an emerging treatment strategy for rare cancers such as urachal (UrC) and primary bladder adenocarcinoma (PBAC). In this study, we performed a multicentre sample collection and developed a DNA sequencing (161 gene Oncomine Comprehensive Assay Panel) and data processing pipeline for the detection and therapeutic interpretation of targetable genetic alterations in these two rare cancer entities. Our analyses revealed actionable alterations (e.g. *EGFR*, *BRCA*, *CCND1/2/3*, *ERBB2*, *MET* and *METex14*) in a high rate of cases, suggesting this approach to be feasible for both UrC and PBAC.

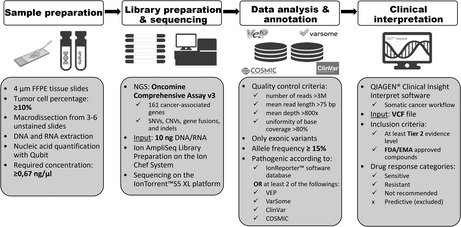

## 1 INTRODUCTION

According to various definitions, cancers with annual incidence rates of 2–15 cases per 100,000 persons are considered rare.^1^ Rare tumours account for more than 20% of all reported cancers, which is higher than the most commonly occurring single tumour type.^2^, ^3^ Rare cancers face specific challenges such as late and often incorrect diagnosis and lack of clinical expertise, research interest and standard treatments.^3^ Consequently, the average survival time of patients with rare cancers is lower than that of patients with more common malignancies.^2^

While urothelial carcinoma of the bladder is a common malignancy, other histological types, such as primary adenocarcinoma of the bladder (PBAC), account for <1% of newly diagnosed bladder cancers. PBAC is derived from the urothelium but exhibits a pure glandular phenotype.^4^ Although the urachal remnant is not a direct anatomical component of the urinary bladder, urachal adenocarcinoma (UrC) is usually described together with bladder adenocarcinoma, as it is detected in most cases after invasion into the bladder.^4^ UrC is an aggressive malignancy deriving from the embryological remnant of the urogenital sinus and allantois. It represents an extremely low proportion of bladder cancers with an incidence of ~0.3/100,000,000.^2^, ^5^, ^6^ UrC shares histological and molecular features with colorectal adenocarcinoma (CRC), potentially reflecting their similar embryological origin from the cloaca.^7^ As UrC is commonly diagnosed at an advanced stage, up to 50% of patients require systemic treatment.^8^

Currently, there are no standard evidence‐based guidelines for the management of PBAC and UrC. According to small retrospective studies and case reports on UrC, 5‐FU‐based treatments seem to provide superior response rates compared to platinum‐based therapies, while 5‐FU/platinum combinations showed the best oncological results, although with the highest toxicity.^8^, ^9^ Owing to the lack of clinically proven systemic therapies, targeted treatments based on genomic profiling and biological rationale represent a promising personalized strategy for rare cancers. However, for UrC and PBAC, only a few published series are available relating to targeted therapies.^10^, ^11^, ^12^, ^13^, ^14^

From the perspective of treatment personalization, it is essential to understand the molecular mechanisms as well as the genetic alterations driving tumour development and conferring responses to specific therapies. In the past few years, the mutational pattern of UrC has been intensively investigated, although the number of examined cases and genes remains limited. Most studies have focused on genes in the RAS/PI3K signalling pathway and frequently found recurring mutations in the *KRAS* gene. In addition, *NF1*, *GNAS*, *NRAS* and *PIK3CA* mutations have also been recurrently detected.^10^, ^11^, ^15^, ^16^, ^17^, ^18^, ^19^ Considering these overlapping alterations, the genomic background of UrC appears similar to that of CRC. On the other hand, we also found some characteristic differences, as the *APC* gene was much less frequently affected in UrC (10%) than in CRC (80%). In addition, microsatellite instability can be detected in 15% of CRCs but is rarely found in UrC.^20^ Based on these findings, UrC represents a similar but clearly distinct molecular pattern compared with CRC.

Much less data are available on the genetic background of PBAC, showing alterations mainly in the MAPK or Wnt pathway genes.^16^, ^21^ Therefore, further investigation is needed to gain a more detailed insight into the molecular background of both UrC and PBAC. An additional missing step towards the clinical implementation of genomic profiling is the lack of a systematic approach for interpreting its potential to guide therapeutic intervention. Thus, in this multicentre study, we performed a genomic analysis of UrC and PBAC samples using a large, commercially available next‐generation sequencing panel with 161 cancer‐related genes. In addition, to identify potentially effective drugs, clinical interpretation was performed using an evidence‐based decision support tool.

## 2 MATERIALS AND METHODS

### 2.1 Clinical samples and data collection

Formalin‐fixed paraffin‐embedded (FFPE) tumour tissues from 41 UrC and 13 PBAC patients were retrospectively collected from nine academic centres. Clinicopathological characteristics, including age, sex, tumour localization, Sheldon/Mayo stage, grade, lymph node status, presence of distant metastasis and survival outcomes, were retrieved from medical records using a standardized datasheet. Histopathology slides of all cases were reviewed and verified in accordance with World Health Organization (WHO) criteria by a genitourinary pathologist (H.R.). The study conformed to the Declaration of Helsinki and the institutional ethics committee approved the study protocol (SE TUKEB 74/2016).

### 2.2 Nucleic acid extraction and next‐generation sequencing (NGS)

Samples from either radical/partial cystectomy or transurethral resection of the bladder (TURB) were processed for next‐generation sequencing. Tumour DNA and RNA were extracted from 4 μm‐thick FFPE tissue slides. Macrodissection was performed to minimize the contamination with non‐malignant tissues. For this, a board‐certified genitourinary pathologist (H.R.) marked the tumour areas on haematoxylin and eosin (H&E)‐stained tissue slides. Corresponding areas were scraped carefully, DNA was extracted using High Pure PCR Template Preparation Kit (Roche, Mannheim, Germany), and RNA was isolated using MagMAX™ FFPE DNA/RNA Ultra Kit (Thermo Fisher Scientific, Waltham, MA, USA) according to the manufacturer's recommendations. Extracted nucleic acid concentration was quantified with Qubit™ dsDNA and RNA High‐Sensitive Assay kit (Thermo Fisher Scientific, Waltham, MA, USA) on the Qubit fluorometer (Thermo Fisher Scientific, Waltham, MA, USA).

We used an amplicon‐based targeted next‐generation sequencing (NGS) assay (Oncomine Comprehensive Assay v3, OCAv3, Thermo Fisher Scientific, Waltham, MA, USA) to identify relevant single nucleotide variants (SNVs), copy number variants (CNVs), gene fusions and small insertions and deletions (indels) from 161 unique genes. NGS library preparation was performed using Ion AmpliSeq Library Preparation on the Ion Chef System (Thermo Fisher Scientific, Waltham, MA, USA). The PCR amplification was performed using an input nucleic acid concentration of 10 ng. Sequencing was performed on the IonTorrentS5 XL platform, according to the manufacturer's protocol. The average coverage of all the runs was approximately 2500x. Ion Reporter™ software (Thermo Fisher Scientific, Waltham, MA, USA) was used for the initial automated analysis. Sequencing reads were aligned with human genome assembly 19 (hg19) and embedded as the standard reference genome in the software. Coverage analysis reports from the Ion Reporter™ Software, which provides measurements of mapped reads, mean depth, uniformity and alignment over a target region, were used to assess the quality of the sequencing reactions. Sample quality control (QC) was performed according to the following criteria: total number of reads >3 M, mean read length >75 bp, mean depth >800x and uniformity of base coverage >80%. Exonic variants detected at an allele frequency of >15% were filtered and annotated using the database provided in the Ion Reporter™ software, and variants were checked in four freely available online databases (Ensembl Variant Effect Predictor (VEP)/ClinVar/VarSome/COSMIC) to identify pathogenic variants. A variant was considered a pathogenic alteration when the Ion Reporter™ software and/or two external databases described it as a pathogenic/likely pathogenic mutation.

### 2.3 Clinical interpretation of sequencing data

**FIGURE 1:**
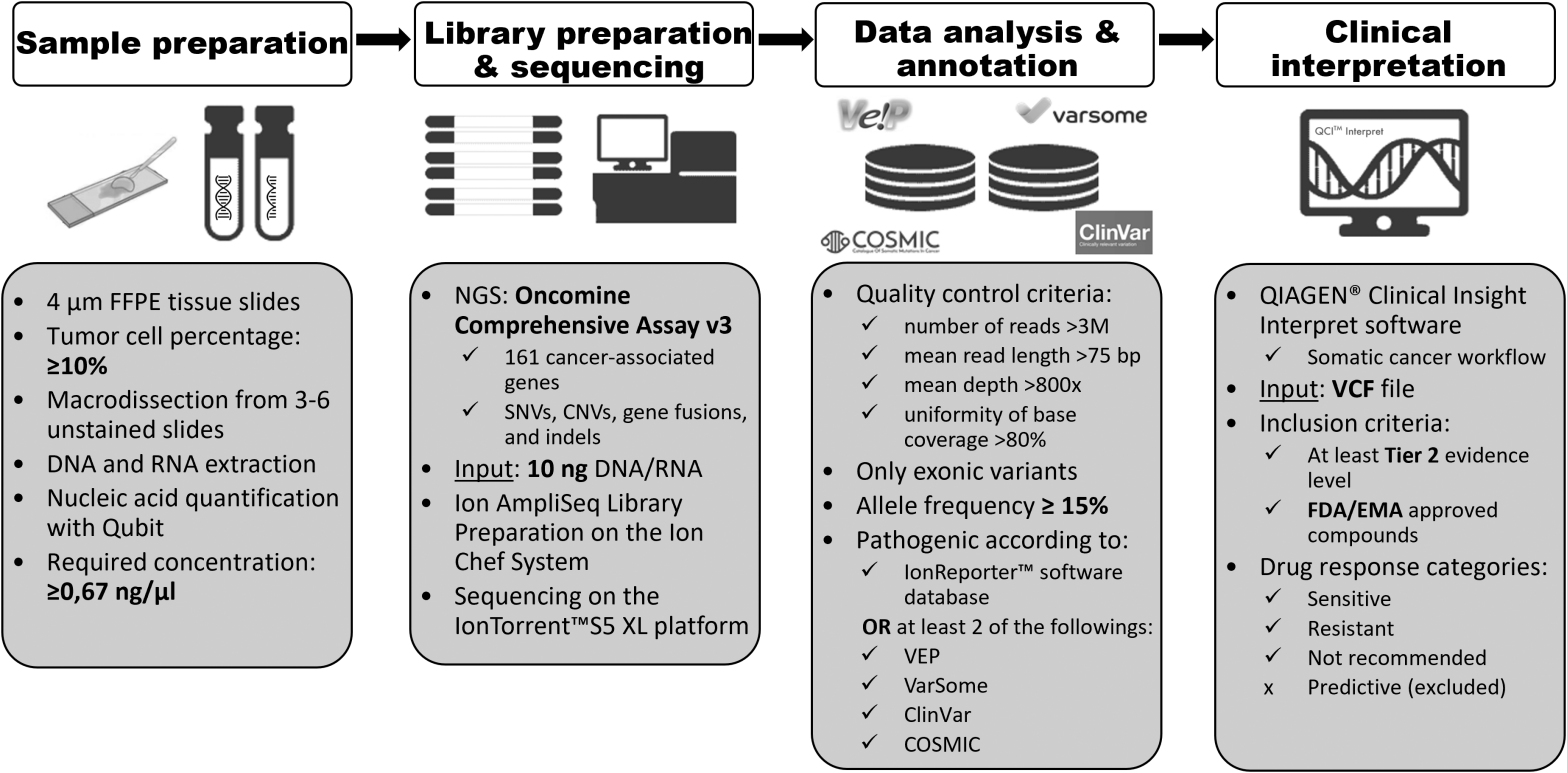
Methodological pipeline for identification of therapeutic targets and drugs for UrC and PBAC.

The therapeutic relevance of pathogenic variants was evaluated using an evidence‐based decision support software, QIAGEN® Clinical Insight Interpret (QCI®, Qiagen, Hilden, Germany) which assesses genomic variants from a therapeutic perspective in the context of published biomedical literature, professional association guidelines, publicly available databases, drug labels and clinical trials. As the software works with curated information from several relevant sources, the list of drugs is constantly expanding. The database search was performed on 16 December 2021. The somatic cancer workflow was used to match each patient's molecular profile to relevant therapeutic information. The software automatically computed actionability classifications (Tier 1–3) for each alteration according to the AMP/ASCO/CAP guidelines (Table S1). In addition, it provides recommendations and contraindications by categorizing drug‐mutation interactions as ‘sensitive’, ‘resistant’, ‘not recommended’ or ‘predictive’. For this study, we considered sensitive, resistant and not recommended drug‐mutation interactions but excluded the category called ‘predictive’ and focused only on FDA/EMA‐approved drugs. Since no Tier 1 recommendations exist for UrC and PBAC and we excluded recommendations with a low evidence level (Tier 3), only variants with Tier 2 recommendations were included (Figure 1).

## 3 RESULTS

### 3.1 Cohort characteristics

Forty‐one patients with UrC and 13 patients with PBAC were included in this study. A full description of patient characteristics is shown in Table 1.

**TABLE 1:** Clinicopathological characteristics of UrC and PBAC patients

		UrC cohort		PBAC cohort	
		*n* = 41	%	*n* = 13	%
Age (year)	Median (Range)	49.5 (22–77)	‐	59 (38–75)	‐
Sex	Male	22	53.7	8	61.5
	Female	19	46.3	5	38.5
Haematuria	Yes	28	84.8	5	83.3
	No	5	15.2	1	16.7
	NA	8	‐	7	‐
Abdominal pain	Yes	2	6.5	2	28.6
	No	29	93.5	5	71.4
	NA	10	‐	6	
Palpable tumour	Yes	1	3.2	1	14.3
	No	30	96.8	6	85.7
	NA	10	‐	6	‐
UrC type	Intestinal	18	46.2	17	53.1
	Mucinous	12	30.8	9	28.1
	NOS	5	12.8	4	12.5
	SRC	2	5.1	2	6.3
	Mixed	2	5.1	‐	‐
	NA	2	‐	9	‐
Calcification	Yes	2	5.4	‐	‐
	No	35	94.6	‐	‐
	NA	4	‐	‐	‐
Signet ring cell component	Yes	‐	‐	3	25.0
	No	‐	‐	9	75.0
	NA	‐	‐	1	‐
Sheldon staging	I	0	0.0	‐	‐
	II	0	0.0	‐	‐
	IIIA	28	70.0	‐	‐
	IIIB	1	2.5	‐	‐
	IIIC	1	2.5	‐	‐
	IIID	0	0.0	‐	‐
	IVA	3	7.5	‐	‐
	IVB	7	17.5	‐	‐
	NA	1	‐	‐	‐
Mayo staging	I	10	25.6	1	9.1
	II	19	48.7	6	54.5
	III	3	7.7	4	36.4
	IV	7	18.0	‐	‐
	NA	2	‐	2	‐
Pathological stage	T1	‐	‐	0	0.0
	T2	‐	‐	3	23.1
	T3	‐	‐	5	38.5
	T4	‐	‐	5	38.5
Grade	I	‐	‐	1	9.1
	II	‐	‐	6	54.5
	III	‐	‐	4	36.4
	NA	‐	‐	2	‐
LN status	LN−/LNx	19	76.0	8	61.5
	LN+	6	24.0	5	38.5
	NA	16	‐	‐	‐
M status	M−	28	77.8	‐	‐
	M+	8	22.2	‐	‐
	NA	5	‐	‐	‐
LN/M status	LN/M+	11	26.8	7	53.8
	LN/M−/LN/Mx	30	73.2	6	46.2
Surgery	TURB	1	2.7	0	0.0
	Partial CE	28	75.7	1	7.7
	Radical CE	8	21.6	12	92.3
	NA	4	‐	‐	‐
Umbilectomy	Yes	21	63.6	‐	‐
	No	12	36.4	‐	‐
	NA	8	‐	‐	‐

Abbreviations: *CE* cystectomy, *LN* lymph node, *LN+* positive lymph node status, *LN−* negative lymph node status, *LNx* unknown lymph node status, *M+* positive distant metastatic status, *M−* negative distant metastatic status, *Mx* unknown distant metastatic status, *NA*. not available, *NOS* not otherwise specified, *SRC* signet ring cell carcinoma, *TURB* transurethral resection of bladder.

### 3.2 Genomic alterations detected by NGS

**FIGURE 2:**
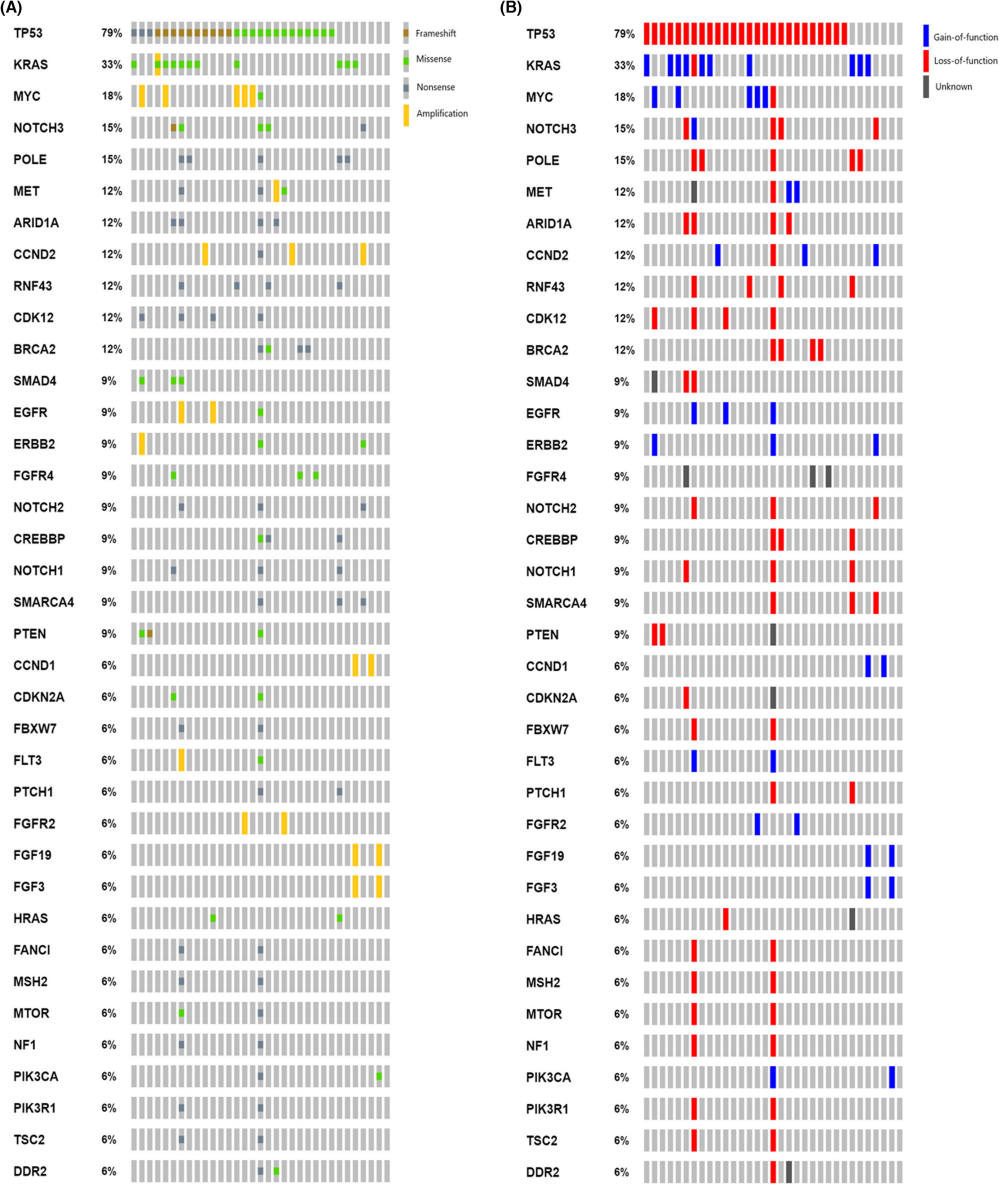
Oncoprint presentation of recurrently mutated genes in UrC by the type (A) and functional effect (B) of alterations.

**FIGURE 3:**
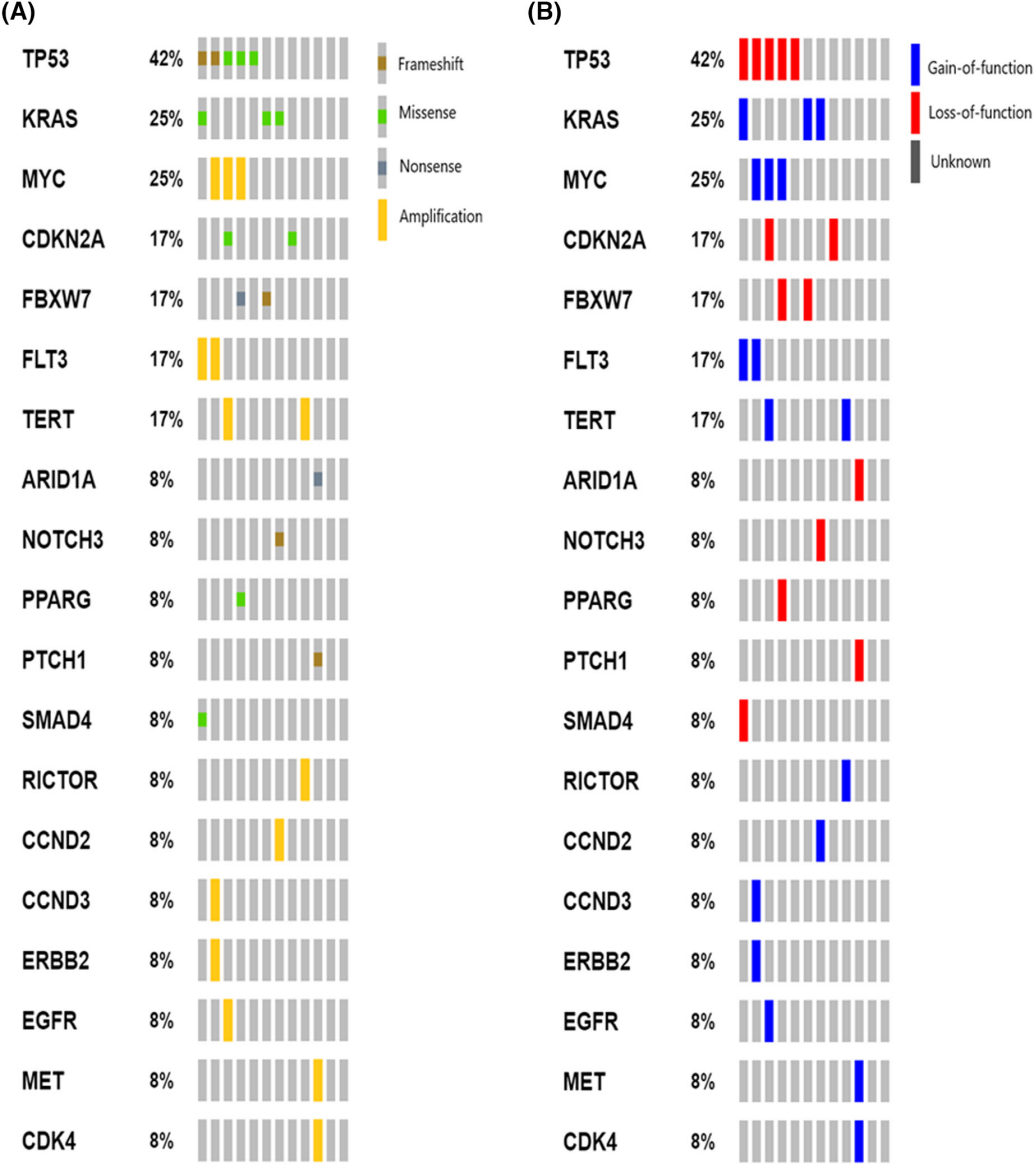
Oncoprint presentation of recurrently mutated genes in PBAC by the type (A) and functional effect (B) of alterations.

Eighty percent (33/41) of UrC and 92% (12/13) of PBAC samples passed sequencing quality control. The mutational patterns of UrC and PBAC are presented in Figures 2 and 3 respectively. Our sequencing analyses revealed 191 pathogenic SNVs in 68 genes (176 in the UrC and 18 in the PBAC cohort), including 89 missense mutations, 87 nonsense mutations, nine frameshift deletions and six frameshift insertions. Ion Reporter™ software and manual annotation were concordant in 63% (121/191) of the variants. Most SNVs were loss‐of‐function alterations (74% for UrC and 83% for PBAC). The most frequently affected genes in both UrC and PBAC were *TP53* (UrC, 79%; PBAC, 42%) and *KRAS* (UrC, 33%; PBAC, 25%).

Amplifications were observed in 16 and 10 genes in the UrC and PBAC cohorts respectively. *MYC* amplification was the most frequently identified CNV in both groups (UrC: 15%, PBAC: 25%).

In the UrC cohort, 37 genes were recurrently affected (in at least two patients). However, only seven recurrently affected genes were detected in PBAC.

RNA was analyzed in 24 UrC and 10 PBAC samples. Due to the low quality of the input RNA, the sequencing results of two UrC samples proved to be invalid. The only alteration detected at the RNA level was the *MET* exon 14 skipping alteration, which was present in two UrC and one PBAC samples.

Alterations at the pathway level provide more functional insights. Therefore, we assigned the examined genes to different pathways according to the Vogelstein classification^22^ (Table 2). As *TP53*, the most frequently altered gene, was assigned to both the cell cycle and DNA damage control pathways, these proved to be the most affected pathways, followed by the RAS and PIK3 (MAPK) pathways. More than 90% (30/33) of UrC patients and 67% (8/12) of PBAC patients carried mutations in one or more genes assigned to the cell cycle pathway.

**TABLE 2:** Affected pathways in UrC and PBAC according to Vogelstein's classification

Vogelstein's pathways	Genes	Patients with mutation *n* (%)	
		UrC	PBAC
Cell cycle	*CCND1*; *CCND2*; *CCND3*; *CDKN2A*; *CDK4*; *CHEK2*; *MDM2*; *MYC*; *MYCL*; *MYD88*; *PPP2R1A*; TP53*	30 (91%)	8 (67%)
DNA damage control	*ATM*; *BRCA1*; *BRCA2*; *FANCA*; *FANCD2*; *FANCI*; *MLH1*; *MSH2*; *MSH6*; *PALB2*; TP53*	26 (79%)	5 (42%)
RAS	*BRAF*; EGFR*; ERBB2*; FGFR2*; FGFR3*; FGFR4*; FLT3*; GNAQ*; GNAS*; *HRAS*; *KRAS*; *MAP2K1*; MET*; *NF1*; *NRAS*; RET*	20 (61%)	6 (50%)
PI3K	*AKT1*; EGFR*; ERBB2*; FGFR2*; FGFR3*; FGFR4*; FLT3*; GNAQ*; GNAS*; MET*; *PIK3CA*; *PIK3R1*; *PTEN*; RET*; *TSC2*	14 (42%)	4 (33%)
NOTCH	*FBXW7*; *NOTCH1*; *NOTCH2*; *NOTCH3*	6 (18%)	3 (25%)
TGF‐β	GNAS*; *SMAD4*	4 (12%)	1 (8%)

*Note*: *Gene assigned to more than one pathway.

### 3.3 Therapeutic interpretation / In silico therapy prediction

**FIGURE 4:**
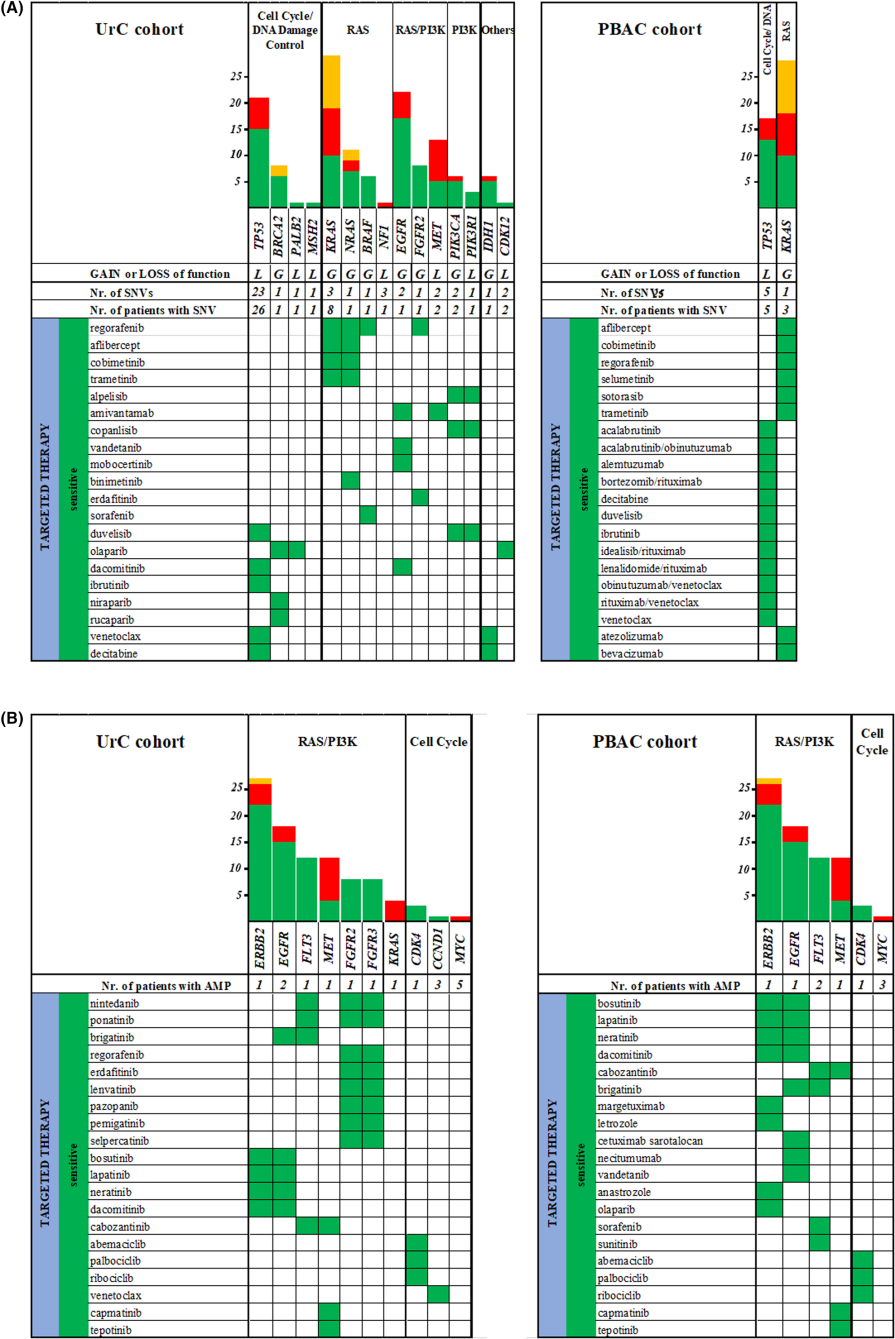
Most recommended targeted therapeutic agents for A) SNVs and B) CNVs.

We used the QCI® software to search for targeted and chemotherapy recommendations and contraindications for FDA/EMA‐approved drugs with at least Tier 2 evidence level. Below, we only describe the sensitive (recommended) mutation‐drug combinations, while resistant and not recommended combinations are given in Table S2. FDA‐and/or EMA‐approved therapies were found in 31 (94%) patients with UrC and eight (67%) with PBAC. Notably, none of these drugs has been approved for the treatment of patients with UrC or PBAC. In the UrC cohort, 53 targeted therapeutic agents and 12 chemotherapeutic agents were listed as the recommended drugs based on SNVs. In the PBAC cohort, 18 potentially effective targeted therapeutics and six chemotherapeutic compounds were reported based on alterations in *TP53* and *KRAS* genes (Figure 4). The therapeutic recommendations for individual patients are presented in Table S2.

When investigating the involvement of different pathways, we found that the majority of the recommended agents for both UrC and PBAC targeted members of the RAS/PIK3 pathways. Regorafenib, a multi‐kinase inhibitor, was recommended for mutations in four RAS pathway genes (*KRAS*, *NRAS*, *BRAF* and *FGFR2*) (Figure 4A).

Therapeutic recommendations based on gene amplification were also considered. Ten of the 19 amplified genes (*CCND1*, *CDK4*, *EGFR*, *ERBB2*, *FGFR2*, *FGFR3*, *FLT3*, *KRAS*, *MET* and *MYC*) were relevant for therapy prediction. Drugs were recommended for 11 (33%) patients with UrC and four (33%) with PBAC based on their CNVs. Forty‐six targeted therapeutic agents and five chemotherapeutic compounds were assigned based on their sensitive alteration‐drug interactions. As with SNVs, the majority of recommended drugs based on copy number gains were RAS/PIK3 pathway inhibitors, mainly multi‐kinase inhibitors (e.g. nintedabin and ponatinib) (Figure 4B). The therapeutic recommendations for individual patients are presented in Table S2.

## 4 DISCUSSION

PBAC and UrC are rare and aggressive malignancies, with a median survival of 12–24 months for locally advanced or metastatic disease.^9^ This poor prognosis is the result of many different factors such as (1) delayed symptoms resulting in diagnosis at advanced tumour stages, (2) no standard‐of‐care therapeutic recommendations and (3) poorly known molecular pathogenesis and genomic landscape of the tumour.^23^ As randomized trials for the evaluation of the clinical benefits of various drugs in UrC and PBAC are not feasible, precision medicine is of prominent therapeutic interest for these rare cancers.^24^ Therefore, comprehensive genome profiling may be fundamental for driving therapeutic decisions in these patients. In the present study, we performed mutational analyses of tumour tissues from patients with UrC and PBAC and sought to identify targetable alterations and corresponding drugs that could potentially be effective for these patients.

In line with previous reports, our genomic analysis found *TP53* (UrC: 79%, PBAC: 42%) to be the most commonly affected gene in both UrC and PBAC.^10^, ^15^, ^16^, ^18^, ^19^, ^21^ As *TP53* mutated tumours progress faster and respond poorly to anticancer therapy, targeting p53 for cancer therapy seems to be an attractive strategy.^25^ Although *TP53* has previously been considered undruggable owing to its essential role in cell survival, many drugs targeting *TP53* mutant tumours have been tested in early phase (Phase I/II) clinical trials.^26^

*KRAS* mutations in UrC have been extensively investigated, as it is a commonly affected oncogene in CRC, a tumour type sharing large histological and molecular similarities with UrC. After summarizing the published literature on the prevalence of *KRAS* mutations in both UrC and PBAC, it proved to be the most frequently tested gene with alterations in ~30% of UrC and 25% of PBAC cases, which is in agreement with our present results showing a 33% mutational frequency in UrC and 25% in PBAC.^11^, ^15^, ^16^, ^18^, ^19^ These data underscore the importance of the RAS pathway in both UrC and PBAC.^16^, ^18^, ^21^, ^27^ The vast majority (11/14) of *KRAS* mutations were missense mutations in codon 12 (G12V, G12D and G12A) which is a pattern similar to that found in CRC.^28^ In recent years, several structural and mechanistic studies have led to the clinical development of selective *KRAS* inhibitors. Last year, the FDA granted accelerated approval for sotorasib, the first *KRAS*‐blocking drug for patients with non‐small cell lung cancer (NSCLC). Phase I/II studies (e.g. NCT03600883 and NCT04699188) are currently investigating the efficacy of sotorasib and other G12C‐inhibitors in other tumour types.

In our UrC cohort, four patients carried loss‐of‐function alterations in *BRCA2*. When considering the therapeutic significance of these alterations, only one patient had a potentially significant (Tier 2) SNV, while the rest were categorized as Tier 3. Recently, PARP inhibitors (olaparib, rucaparib and niraparib) have become available for patients with alterations in *BRCA1/2* or other homologous recombinant repair genes. Accordingly, our drug prediction recommended PARP inhibitors for UrC patients with the Tier 2 mutation. However, little is known about the efficacy of PARP inhibitors in UrC patients. The only report on the use of a PARP inhibitor for the treatment of an UrC patient came from a Japanese phase I dose escalation study of niraparib and described progression during therapy. As *BRCA* positivity was not an inclusion criterion in the study, the *BRCA* status of patients with UrC is unknown.^13^

When considering copy number alterations, *MYC* amplification was detected at the highest frequency (5/33, 15%) in our UrC cohort, which was lower than that reported in a previous study (6/17, 35%). In addition, we found *EGFR* amplification in 6% (2/33) of our UrC patients, which was lower than the frequency of 20% reported by Lee et al.^19^ Furthermore, we found recurrent copy number gains in members of the FGF/FGFR signalling pathway (in four UrC and six PBAC patients). Although previous genomic analyses of PBAC did not reveal amplification of *MYC* gene, *MYC* was found to be the most frequently amplified gene (25%) in our study.

MYC is a global transcription factor and a driver of many human malignancies and has proven to be difficult to inhibit directly.^29^ In this context, it is interesting that *CDK12* was found to be a synthetic lethal gene with *MYC*. These findings were corroborated by an independent study demonstrating that CDK inhibition triggered massive downregulation of *MYC* expression and its related genes.^30^ The overlap between *MYC* and the known cellular functions of *CDK12*, as well as the requirement of *CDK12* for optimal processing of *MYC*, collectively indicates *CDK12* is a potential therapeutic target for *MYC*‐dependent cancers.^31^

EGFR is a widely used therapeutic target. Numerous anti‐EGFR compounds, including tyrosine kinase inhibitors (TKIs) and monoclonal antibodies, have been developed and approved for different cancers.^32^ This was also reflected in our results, which identified the second highest number of recommended drugs for patients with tumours harbouring *EGFR* amplification. In the literature, two UrC patients have been reported to receive EGFR inhibitors. One patient with *EGFR* amplification experienced a persistent partial response to cetuximab in the third‐line setting,^10^ while another UrC patient with immunohistochemically proven EGFR overexpression experienced a transient 55% decrease in tumour size with gefitinib treatment.^14^ These results suggest *EGFR* is a potent therapeutic target for UrC treatment.

In addition, we found for the first time that D‐type cyclin (*CCND1/2/3*) genes were affected by activating mutations in both UrC and PBAC samples, with frequencies of 15% and 25% respectively. CCNDs promote cell cycle progression from G1 to S phase by binding to and activating the cyclin‐dependent kinases CDK4/CDK6, thereby imparting oncogenic properties. The cyclin D‐CDK4/6 complex is often hyperactivated in various tumours (e.g. NSCLC, head and neck, renal cell, breast, pancreatic and colorectal cancers), partly by gene amplification, and is therefore an attractive therapeutic target.^33^, ^34^ Recently, multiple CDK4/6 inhibitors have been approved for the treatment of breast cancer.^33^ In addition, several ongoing clinical trials are assessing the efficacy of palbociclib, abemaciclib and ribociclib in other cancers (e.g. NCT03446157, NCT02022982 and NCT03356223). Although molecular alterations in cell cycle pathway genes are not mandatory for the prescription of these drugs, their presence suggests a favourable effect. Accordingly, this mutation‐drug association is being tested in an ongoing phase II pan‐cancer trial (NCT04439201) to assess the efficacy of palbociclib in patients with various malignancies harbouring *CCND1/2/3* amplifications. We have no information about the inclusion of UrC or PBAC patients in the above‐mentioned trial, as preliminary results have not yet been posted. However, based on our results, UrC and PBAC patients carrying activating amplification in their *CCND* genes may be good candidates for future studies.

Our analyses identified one UrC and one PBAC patient with MET amplification. MET alterations, in addition to their primary cancer driver role, can mediate resistance to other targeted therapies, such as EGFR inhibitors. They do this by activating downstream signal transduction, which leads to escape from therapy‐induced cell death.^35^ This effect was reflected in our QCI® drug prediction as it suggested resistant association between MET amplification and anti‐EGFR drugs.

According to recent data, not only the above‐mentioned amplification of MET, but also its exon 14 skipping alteration (METex14) is associated with acquired resistance to EGFR‐targeting compounds.^36^ METex14 has been identified in approximately 3% of lung NSCLCs and other solid tumours such as breast cancer and glioblastoma. In 2020 and 2021, two MET inhibitors, capmatinib and tepotinib, were approved for use as monotherapies in patients with NSCLC carrying METex14.^37^ To our knowledge, this is the first report of METex14 alterations in treatment‐naïve UrC and PBAC, with an overall incidence of 3/38 (7%–8%). Considering the durable response observed in NSCLC patients with METex14 alterations, UrC and PBAC patients with these alterations may also benefit from tepotinib or capmatinib therapy. Accordingly, in the only published UrC patient treated with tepotinib, durable disease stabilization was observed.^12^

We identified copy number gain of ERBB2 (HER2) in one UrC and one PBAC sample. In breast cancer, ERBB2 amplification is a well‐known prognostic biomarker of poor survival in the absence of anti‐HER2 therapy.^38^ There is an abundance of approved HER2‐targeted agents not only for breast cancer, but also for other cancer entities, such as metastatic gastric or gastroesophageal junction cancers.^39^ Currently, several ongoing clinical trials are evaluating the potential benefits of targeting HER2 in various tumour types (e.g. NCT02465060 and NCT02675829). Little is known about the prevalence of ERBB2 amplification in patients with UrC or PBAC. A study investigating the prevalence of ERBB2 amplification in different tumours identified ERBB2 amplification in 2 of 7 (28%) UrC samples.^39^ This study also showed the clinical benefits of HER2‐targeted therapy in tumours for which HER2‐inhibitors have not yet been approved.^39^ In the present study, the QCI® drug prediction algorithm recommended the highest number of drugs for ERBB2 amplified tumours, suggesting that this alteration is an attractive therapeutic target.

FLT3 amplification might also be a potentially actionable molecular alteration, although the majority of kinase inhibitors approved so far are relatively nonspecific for FLT3 (e.g. nintedanib, ponatinib, sorafenib and sunitinib). The off‐target activities of these multi‐kinase inhibitors can contribute to higher toxicity, causing severe adverse events.^40^ Accordingly, in the case report by Loh et al.,^11^ an UrC patient with FLT3 amplification received sorafenib therapy, but it had to be discontinued shortly after drug initiation due to a serious adverse event. Sunitinib was subsequently administered without toxicity; however, an additional treatment change was required because of disease progression. Next‐generation inhibitors, such as quizartinib or gilteritinib, are more specific and potent FLT3 inhibitors with more favourable toxicity profiles; however, to date, these drugs are approved only for acute myeloid leukaemia, and thus no data on their activity in solid tumours are available.^41^

This study has several strengths. This is one of the largest studies on UrC and PBAC with respect to the number of cases and assessed genes. This is the first study on UrC and PBAC that systematically applies a clinical decision support tool to match driver aberrations with clinically approved drugs. Finally, we report here for the first‐time recurrent alterations in *CCND1‐2*, *NOTCH3*, *RNF43*, *CDK12*, *FGFR4*, *CREBBP* and *SMARCA4* in UrC and mutations in *MYC*, *CDKN2A* and *FLT3* in PBAC.

On the other hand, this study has several limitations. Due to the rarity of UrC and PBAC, a retrospective approach was needed to collect samples from multiple institutions over a long time period. Associated differences in sample handling and specimen age may result in heterogeneous quality of FFPE tissues. Consequently, a relatively high rate (~9%) of the samples did not pass the quality control. In addition, as the clinical interpretation could be carried out in a retrospective manner, we were not able to assess whether the recommended drugs would have been effective in the assessed patients with UrC and PBAC. A further limitation is the heterogeneity of databases regarding both the judgement of pathogenicity and druggability of certain variants. In addition, as the CNVs were predicted by bioinformatics tools, orthogonal validation, for example, by *fluorescence* in‐situ hybridization (FISH), would be needed to validate the presence of the alteration. Some of these limitations could be addressed by the addition of in vitro (e.g. organoid) and in vivo (patient‐derived tumour xenografts) models to improve our ability to predict drug responses, which could then improve treatment selection for patients with rare cancers.

In conclusion, our results suggest significant overlaps in the genomic landscapes of UrC and PBAC. The cell cycle pathway was the most affected pathway, followed by the DNA damage control, RAS and PI3K pathways. However, a large individual heterogeneity was observed in the mutation patterns. In most cases, at least one potentially druggable alteration was identified, highlighting the potential of genetic profiling to guide the treatment of these rare malignancies.

## AUTHOR CONTRIBUTIONS

**Melinda Varadi:** Data curation (equal); investigation (equal); project administration (equal); visualization (equal); writing – original draft (lead). **Nikolett Nagy:** Data curation (lead); investigation (equal); project administration (equal); visualization (equal); writing – original draft (equal). **Henning Reis:** Data curation (equal); investigation (equal); writing – review and editing (equal). **Boris Hadaschik:** Data curation (equal); writing – review and editing (equal). **Christian Niedworok:** Data curation (equal); writing – review and editing (equal). **Orsolya Modos:** Data curation (equal); project administration (equal); writing – review and editing (equal). **Attila Szendroi:** Data curation (equal). **Jason Ablat:** Data curation (equal); writing – review and editing (equal). **Peter Black:** Data curation (equal); writing – review and editing (equal). **David Keresztes:** Data curation (equal); project administration (equal); writing – review and editing (equal). **Anita Csizmarik:** Data curation (equal); project administration (equal); writing – review and editing (equal). **Csilla Olah:** Data curation (equal); writing – review and editing (equal). **Nadine T Gaisa:** Data curation (equal); writing – review and editing (equal). **Andras Kiss:** Data curation (equal). **Jozsef Timar:** Data curation (equal). **Erika Toth:** Data curation (equal); methodology (equal). **Erzsébet Csernak:** Investigation (equal); methodology (equal). **Arpad Gerstner:** Resources (equal); software (equal). **Vinay Mittal:** Software (equal); validation (equal); visualization (equal). **Sofia Karkampouna:** Data curation (equal); investigation (equal). **M Kruithof‐de Julio:** Data curation (equal); writing – review and editing (equal). **Balazs Gyorffy:** Data curation (equal); formal analysis (equal); writing – review and editing (equal). **Gabor Bedics:** Data curation (equal); software (equal). **M. Rink:** Data curation (equal); writing – review and editing (equal). **Margit Fisch:** Data curation (equal); writing – review and editing (equal). **Pèter Nyiràdy:** Data curation (equal); writing – review and editing (equal).

## FUNDING INFORMATION

T. S. was supported by a János Bolyai Research Scholarship of the Hungarian Academy of Sciences (BO/00451/20/5) and by the New National Excellence Program (ÚNKP‐21‐5‐SE‐3) and the K139059 grant of the Ministry for Innovation and Technology from the source of the National Research Development and Innovation Fund. The project has also received funding from the EU's Horizon 2020 research and innovation program under grant agreement No. 739593 and from the Ministry for Innovation and Technology of Hungary from the National Research, Development and Innovation Fund, financed under the TKP2021‐EGA‐24 and TKP2021‐NVA‐15 funding schemes.

## CONFLICT OF INTEREST

The authors declare no potential conflicts of interest.

## ETHICS STATEMENT

The study conformed to the Declaration of Helsinki and the institutional ethics committee approved the study protocol (SE TUKEB 74/2016).

## Supporting information


Table S1

Table S2

## Data Availability

The data generated in this study are available within the article and its supplementary data files.
