# Supplementary material for: Clinical sequencing identifies potential actionable alterations in a high rate of urachal and primary bladder adenocarcinomas
Source: Cancer Med. 2023 Jan 20;12(7):9041–54. doi: 10.1002/cam4.5639 (PMC10134276; doi:10.1002/cam4.5639)
Supplement: Supplementary file 1 — Table S1 [file CAM4-12-9041-s002.docx]

**SUPPLEMENTARY TABLES**

Supplementary Table S1. Clinical Significance of Variants Based on AMP / ASCO / CAP Guidelines*

| **Strong**  **Significance** | **Tier 1A** | • Biomarker predicts response or resistance to an FDA or EMA approved therapy, according to drug label or professional guidelines for this diagnosis  • Biomarker included in professional guidelines is prognostic or diagnostic for this diagnosis |
| --- | --- | --- |
|  | **Tier 1B** | • Biomarker predicts response or resistance to a therapy for this diagnosis based on well powered studies  • Biomarker is prognostic or diagnostic for this diagnosis based on well-powered studies  • Biomarker is associated with response or resistance to an FDA or EMA approved therapy according to drug label or professional guidelines but only for different diagnosis |
| **Potential**  **Significance** | **Tier 2C** | • Biomarker is an inclusion criterion for an active clinical trial  • Biomarker is prognostic, or diagnostic based on multiple small studies |
|  | **Tier 2D** | • Biomarker shows plausible response or resistance based on case or preclinical studies  • Biomarker may assist in disease diagnosis or prognosis based on small studies |
| **Uncertain**  **Significance** | **Tier 3** | Biomarker has uncertain clinical significance and not known to be likely benign or benign |
| **Adapted from PMID:27993330 jmd.amjpathol.org/article/S1525-1578(16)30223-9/pdf | | |

Supplementary Table S2. Individual patient based therapeutic recommendations and contraindications in UrC and PBAC (Excel sheets)
